# Genome sequences of *pirAB*^+^ and *pirAB*^−^ *Vibrio campbellii* strains isolated from shrimp ponds with mortality outbreaks carry type VI secretion systems

**DOI:** 10.1128/mra.00387-26

**Published:** 2026-08-17

**Authors:** Jenz Contante, Leobert dela Peña, Edgar Amar, Clarissa Gomez, Albert Noblezada, Victor Marco Emmanuel Ferriols, Erh-Min Lai, Chih-Horng Kuo, Mary Nia M. Santos

**Affiliations:** 1 National Fisheries Research and Development Institute 594060, Quezon City, Metro Manila, Philippines; 2 Aquaculture Department, Southeast Asian Fisheries Development Center 69338, Tigbauan, Iloilo, Philippines; 3 Philippine Genome Center Visayas, University of the Philippines Visayas 54730, Miagao, Iloilo, Philippines; 4 Institute of Plant and Microbial Biology, Academia Sinica 38017, Taipei, Taiwan; University of Wisconsin-Madison, Madison, Wisconsin, USA

**Keywords:** *Vibrio campbellii*, aquaculture, shrimp, genome, type VI secretion system

## Abstract

*Vibrio campbellii* strains PH1401 and PH1409 were isolated from shrimp ponds with documented mortality outbreaks in the Philippines. PH1401 and PH1409 share identity with *V. campbellii* strain BoB-53. Whole-genome analysis reveals full-length *pirAB* binary toxin genes in PH1401*.* Both strains carry three type VI secretion systems.

## ANNOUNCEMENT

Acute hepatopancreatic necrosis disease (AHPND) causes severe losses in shrimp-producing countries worldwide (1). AHPND-causing *V. campbellii* strains have been reported (2–5), but relatively few whole-genome sequences are available (2, 6). The strains described here were isolated from the hepatopancreas of symptomatic *Penaeus monodon* from mortality outbreaks in Philippine shrimp farms between 1998 and 2000. PH1401 was isolated from Iloilo (10°48′32.7″N, 122°38′24.6″E), whereas PH1409 was isolated from Negros Occidental (10°42′34.2″N, 122°57′17.5″E). Isolates were cultured on nutrient agar (Merck, Germany) with 1.5% NaCl. Both were initially identified as *Vibrio harveyi* based on biochemical tests (7).

PH1401 was cultured in LB broth with 2.5% NaCl at 30°C. Genomic DNA was extracted with Nanobind HMW DNA kit (PacBio, USA) following the manufacturer’s protocol. Libraries were prepared as described by Salazar et al. (8). Illumina libraries were prepared from DNA sheared to ~350 bp without PCR amplification using the KAPA LTP library preparation kit (KK8232, Roche). Paired-end sequencing on NovaSeq 6000 generated 9,639,510 short reads, totaling 1,455.4 Mbp. Reads were qualified with FastQC v0.11.0 (9) and trimmed with Trimmomatic v0.36.6 (10). For long reads, DNA was purified with AMPure XP and enriched with the SRE XL kit (PacBio, USA). Libraries were prepared using the ligation sequencing kit (SQK-LSK109) with native barcoding expansion 96 (EXP-NBD196) and then sequenced on MinION with R9.4.1 flow cell (FLO-MIN106D). Guppy v6.4.2 basecalling produced 11,333 reads, totaling 295 Mbp. Hybrid *de novo* assembly (Table 1) was executed with Unicycler v0.5.0 (11), with --*mode conservative* and --*min_fasta_length* 200 parameters, and then polished with Pilon v1.20.1 (12). Completeness was quantified with BUSCO v5.8.2 using the *gammaproteobacteria_odb12* lineage data set (13).

**TABLE 1:** Summary of the assembly statistics of two *Vibrio* strains isolated from shrimp farms in the Philippines

Genomic features	PH1401	PH1409
Contigs	6	75
GC content	45.59	45.50
Genome length (bp)	6,037,120	5,712,890
Contig L50	1	6
Contig N50	3,574,424	303,539
Scaffold L50	1	6
Scaffold N50	3,574,424	303,539
Chromosomes	2	
Plasmids	4	
CDS	5,474	5,041
Genome coverage	460×	89×
Assembly level	Complete genome	Contig
RNA genes	173	108
BUSCO scores	99.87%	99.40%

PH1409 was cultured on marine agar at 30°C. Genomic DNA was extracted using the DNeasy Blood & Tissue Kit (Qiagen, Germany) following the optimized protocol (14). Sequencing libraries were prepared using the Illumina DNA Prep kit (Illumina, USA) and sequenced on an Illumina NextSeq 1000 platform (2 × 151 bp), generating 3,983,556 paired-end reads, totaling 546 Mbp. Reads were assessed using FastQC (9), assembled with Unicycler v0.5.1 (11), and evaluated with QUAST v5.3.0 (15) and BUSCO (13).

Both genomes were annotated using NCBI PGAP v6.5 (16). Full-length *pirAB* genes were detected in PH1401. Average nucleotide identity (17) shows PH1401 and PH1409 share 97.51% and 97.21% identity, respectively, with *V. campbellii* BoB-53. Both genomes carry three type VI secretion system gene clusters with highly conserved structural proteins (99.67% identity), and effector/immunity pairs exhibiting low sequence identity despite conserved protein domains (18). These sequences expand *V. campbellii* genomic resources and support investigations into the distribution of *pirAB* within the T6SS1^+^ lineage.

## Data Availability

This genome project has been registered at the National Center for Biotechnology Information (NCBI) under BioProject accession number PRJNA910658. Raw reads for PH1401 were deposited at the NCBI Sequence Read Archive (SRA) under the accession numbers SRX32535839 and SRX32535840 for short and long reads, respectively. Short reads for PH1409 were deposited under SRX32535838. The genome sequence for *V. campbellii* PH1401 has been deposited at GenBank under the accession numbers CM139086–CM139087 (chromosomes) and JBICRK020000003.1–JBICRK020000006.1 (plasmids). The contig-level genome sequence of *V. campbellii* PH1409 has been deposited under the accession numbers JBICRL020000001–JBICRL020000074.
